# Alginate–Polymethacrylate Hybrid Microparticles as Multi-Unit Enteric Drug Carriers for Posaconazole

**DOI:** 10.3390/pharmaceutics18040467

**Published:** 2026-04-11

**Authors:** Katarzyna Kruk, Marta Szekalska, Eliza Wolska, Iwona Misztalewska-Turkowicz, Karolina Halina Markiewicz, Jolanta Magnuszewska, Agnieszka Zofia Wilczewska, Katarzyna Winnicka

**Affiliations:** 1 Department of Pharmaceutical Technology, Medical University of Białystok, Mickiewicza 2C, 15-222 Białystok, Poland; marta.szekalska@umb.edu.pl; 2 Department of Pharmaceutical Technology, Medical University of Gdańsk, Hallera 107, 80-416 Gdańsk, Poland; eliw@gumed.edu.pl; 3 Department of Polymers and Organic Synthesis, Faculty of Chemistry, University of Białystok, Ciołkowskiego 1K, 15-245 Białystok, Poland; i.misztalewska@uwb.edu.pl (I.M.-T.); k.markiewicz@uwb.edu.pl (K.H.M.); jolmag@uwb.edu.pl (J.M.); agawilczuwb@gmail.com (A.Z.W.)

**Keywords:** microparticles, sodium alginate, polymethacrylates, posaconazole, drug carriers, multi-unit enteric carrier, enteric delivery

## Abstract

**Background/Objectives**: Enteric drug forms are developed to delay drug release to avoid drug degradation in the acidic environment of the stomach or to prevent irritation of the stomach mucosa. The bioavailability of posaconazole (POS) after oral administration depends on stomach pH and food intake. Delayed-release tablets and unmodified oral suspension are the POS formulations currently available on the market. The oral suspension formulation is characterized by highly variable bioavailability, which may significantly affect therapy effectiveness. **Methods:** In this study, multi-unit drug forms with delayed and sustained POS release were designed. Polymeric microparticles consisting of sodium alginate (ALG), methacrylic acid–ethyl acrylate copolymer (EUD), or both, were prepared using the spray-drying technique. The formulations that met the pharmacopoeia enteric release standards in the in vitro dissolution test were subjected to further in vitro evaluation via swelling and mucoadhesion assays, an antifungal activity test, attenuated total reflectance–Fourier transform infrared spectroscopy (ATR-FTIR), and thermal analysis. **Results**: It was shown that EUD formulations at concentrations of 5% and 6% provided enteric release, whereas ALG at 1.5% concentration exhibited a sustained, although not delayed, POS release profile. The optimal blended formulations (EAP15–EAP18), comprising 4% EUD with 1.5–2.0% ALG and either 1% or 4% POS, met the pharmacopoeia criteria for enteric dosage forms. Furthermore, these blends demonstrated the most favorable sustained-release profiles in the buffer phase, ranging from 2 to 3 h. The microparticles exhibited beneficial swelling and mucoadhesive properties, which are essential for prolonging contact with the intestinal mucosa; combined with antifungal properties. **Conclusions**: Obtained carrier may provide a promising preliminary basis for developing a multi-unit, sustained-release enteric dosage form for POS and future in vivo investigations.

## 1. Introduction

The therapeutic efficacy of oral drug delivery systems is closely related to the solubility and dissolution rate of the active pharmaceutical ingredient. It is estimated that approximately 70% of current drugs are poorly water-soluble, falling into Class II or IV of the Biopharmaceutical Classification System (BCS) [1]. In these cases, low solubility often requires the administration of higher doses, which can increase the risk of dose-dependent adverse effects [2,3]. Consequently, developing advanced formulation strategies for BCS Class II and IV drugs remains a critical area of pharmaceutical research.

Microparticles are characterized by a high surface-area-to-volume ratio, which provides a larger surface for drug dissolution. Consequently, increased exposure of microparticles to systemic fluids facilitates a more efficient release process, which is essential for enhancing the bioavailability of poorly water-soluble compounds. [4]. Microparticles may also enable targeted drug delivery and provide prolonged or delayed drug release. Microparticles are a type of multi-unit drug delivery system with a spherical shape and a diameter of 1–500 µm; they consist of an active substance homogeneously dispersed in a matrix [5,6]. Polymers employed in microparticle technology include natural compounds, such as alginates, chitosan, hyaluronic acid, gelatin, and pectin, or synthetic compounds, such as polylactic acid, polycaprolactone, polyethylene glycol, or poly(lactic-co-glycolic acid) [4].

Spray drying is one of the methods employed to obtain microparticles. This method involves the atomization of a liquid formulation (solution, suspension, or emulsion) in a stream of hot inert gas. The active substance can be dissolved or suspended (rarely emulsified) in a polymer solution. The liquid formulation is atomized through the needle, the solvent evaporates, and then the solid product deposits in a collection vessel. Brief thermal exposure time renders the spray-drying process safe for thermolabile substances [7,8]. The spray-drying technique is effective as it is characterized by high reproducibility, simplicity, and cost-effectiveness; moreover, it could potentially improve the pharmacokinetics profile of hydrophobic drugs [9].

Sodium alginate (ALG) is a natural polysaccharide polymer with an anionic character that is extracted from brown algae. The ALG chain consists of mannuronic and guluronic acid residues linked by β-1,4-glycosidic bonds. ALG is characterized by high water solubility, gelation ability, inert character, good mucoadhesive properties, non-toxicity, and biocompatibility. Given its beneficial biological properties (safety, chelating capacity, and no accumulation in the body), ALG is registered by the Food and Drug Administration (FDA) for pharmaceutical, biomedical, and food purposes [10]. Due to its chemical properties (ALG precipitates at a low pH < 3) and its ease of gel formation, the polymer may be promising in the design of formulations with delayed and sustained drug release [10,11,12,13,14,15]. ALG is widely employed in micro- and nanoparticle technology since it is combined with other natural or synthetic polymers to modify the release of active substances, such as hydrophilic and hydrophobic drugs [10].

Polymethacrylates are synthetic polymers available under various commercial names consisting of methacrylic acid, methacrylic acid esthers, and dimethylaminoethyl methacrylates in different proportions [16,17]. The primary distinction between these polymers is the dissolution differences under various pH conditions, which render them highly applicable and widely employed in pharmaceutical technology to modify the drug release profile [18]. Eudragit^®^ is a brand of polymethacrylate copolymers that occur as solutions, aqueous suspensions, powders, and granules that are biocompatible, degradable in physiological conditions, and do not cause toxic effects. Eudragit^®^ L 30 D-55 (EUD) is a 30% water suspension, which dissolves above pH 5.5, and is widely applied in the development of enteric drug forms [19]. Polymethacrylates from the Eugragit^®^ family are applied most frequently as tablet or pellet coatings [20,21], although they may also be employed in enteric microparticle or matrix tablet technology [22].

Posaconazole (POS) is an antifungal agent belonging to the azole group. Its mechanism of action consists of the inhibition of ergosterol synthesis, which is a necessary element of fungal cell wall construction. POS demonstrates antifungal properties against a wide range of pathogens, including *Aspergillus*, *Candida*, and *Fusarium* species [23,24]. The main challenge in therapy with POS appears to be its poor and variable bioavailability, which depends on the dosage form, gastric pH, and food intake, and ranges from 8 to 18% for oral suspension to 42–66% for enteric tablets [25]. POS is categorized as Class II in the BCS, with poor solubility and good absorption [26]. Since it is chemically a weak base, it demonstrates higher solubility in acidic gastric conditions. It was shown that lower gastric pH (e.g., after co-administration with an acidic beverage) correlates with increased POS bioavailability [27]. On the contrary, elevated gastric pH, e.g., as a result of receiving proton pump inhibitors or H2-receptor blockers, leads to a decrease in POS solubility, thereby reducing its absorption and bioavailability [28,29,30,31,32]. POS bioavailability after oral administration is also affected by food intake [33]. Notably, delayed-release tablets are characterized by higher and more stable serum concentrations compared with the oral suspension, indicating improved and more consistent bioavailability [34,35]. Despite its lower bioavailability, POS oral suspension is preferred in children since they may be unable to swallow tablets, as well as in seriously ill adults with decreased renal function [28,30]. The aforementioned limitations highlight the need for the development of an enteric drug form suitable for pediatric and geriatric use, which would reduce the variability in drug bioavailability and provide improved efficacy [36]. Recently, a delayed-release oral suspension—a novel drug form for POS—was developed [30,37], which consists of POS and a pH-sensitive polymer combined with a dedicated liquid suspension carrier for oral administration [38]. However, the product does not seem to meet expectations due to the complicated dose preparation process, short shelf life after preparation, and its incompatibility with feeding tubes; moreover, it is currently not available on the market [39]. Despite the documented properties of ALG and EUD, there are only scarce studies concerning drug delivery systems based on an EUD L30 D-55/ALG blended matrix.

The aim of this study was to design POS-loaded microparticles with a blended matrix of sodium alginate and Eudragit^®^ L 30 D-55 that combine the dosage personalization potential of oral suspension with the enteric protection of delayed-release tablets. The developed carriers were evaluated for their swelling and mucoadhesive properties, which are crucial for increasing the intestinal residence time and ensuring prolonged contact with the absorption site. The release kinetics of the carriers were also evaluated.

## 2. Materials and Methods

### 2.1. Materials

Sodium alginate with medium viscosity (1%, 282 mPa·s, 61% mannuronic acid (M), and 39% guluronic acid (G), with an M/G ratio of 1.56) was obtained from Sigma Aldrich (Steinheim, Germany). Eudragit^®^ L 30 D-55 (methacrylic acid–ethyl acrylate copolymer (1:1) dispersion 30%) was received from Evonik Industries AG (Essen, Germany). POS was attained from Kerui Biotechnology Co., Ltd. (Xi’an, China). Methanol was procured from Merck (Darmstadt, Germany). Hydrochloric acid, sodium hydroxide, and sodium phosphate tribasic dodecahydrate were purchased from Chempur (Piekary Śląskie, Poland). Water was distilled using a Milli-Q Reagent Water System (Billerica, MA, USA). Sabouraud dextrose agar was obtained from Biomaxima (Lublin, Poland). Cultures of *Candida albicans* ATCC^®^ 10231, *Candida crusei* ATCC^®^ 6528, and *Candida parapsilosis* ATCC^®^ 22,019 were obtained from the American Type Culture Collection. Nylon membrane filters (0.45 µm) were purchased from Millipore (Billerica, MA, USA). Porcine gastric and small intestine mucosa were received from a veterinary service (Turośń Kościelna, Poland). The porcine intestinal mucosa was prepared by rinsing with 0.9% NaCl. The tissue was partitioned into sections (approx. 2 cm × 2 cm), wrapped to prevent dehydration, and stored at −20 °C for a maximum of one month prior to testing. Before testing, segments were thawed and equilibrated in the dissolution medium for 30 min to ensure proper hydration. Only excerpts demonstrating preserved morphological integrity were selected for this study to ensure data reproducibility. This process did not require the agreement of the Local Ethical Committee for Experiments on Animals. All other reagents utilized in the experiments were of analytical grade.

### 2.2. Development of Polymer Microparticles

Polymer microparticles were prepared using spray-drying technology (mini spray dryer B-290, Büchi, Flawil, Switzerland). The first step was to dissolve EUD in an appropriate amount of water with the experimentally established amount of sodium hydroxide using a magnetic stirrer (Heidolph Instruments, Schabach, Germany). ALG was dissolved in water with the use of a mechanical stirrer (DT 200, Witko, Łódź, Poland). Suitable amounts of the solutions of both polymers were blended in appropriate proportions, and a suitable quantity of POS was homogeneously dispersed in the obtained polymer solutions. The microparticle composition is presented in Table 1. Prepared dispersions were spray-dried under experimentally determined conditions (inlet and outlet temperatures: 150 and 86 °C, respectively; aspirator blower capacity, 85%; pressure, 80 mm Hg; and feed rate, 2.1 mL/min).

### 2.3. Assessment of Microparticle Characteristics

#### 2.3.1. Estimation of POS Loading, Encapsulation Efficacy, and Production Yield

To evaluate POS loading, 10 mg of microparticles was dissolved in phosphate buffer (pH = 6.8) and agitated for 24 h at 75 rpm in a water bath (22 ± 2 °C) and successively diluted with methanol. Each sample was filtered through a 0.45 µm nylon membrane filter and evaluated spectrophotometrically. Blank microparticles were used as a reference to confirm the absence of analytical interference from the polymer matrix.

Drug loading (L) was computed using the following formula:

L = Q_m_/W_m_ × 100 (1)

where Q_m_ is the drug encapsulated in the microparticles, and W_m_ is the microparticle weight.

The mean drug encapsulation efficiency (EE) was calculated using the following expression:

EE = Q_a_/Q_t_ × 100 (2)

where Q_a_ is the actual drug content, and Q_t_ is the theoretical drug content.

The yield of production (Y) was determined by the following formula:

Y = W_m_/W_t_ × 100 (3)

where W_m_ is the microcapsule weight, and W_t_ is the theoretical weight of the drug and polymer.

#### 2.3.2. Particle Size

An optical microscope (Motic BA 400, Moticon, Wetzlar, Germany) was utilized to evaluate the particle size. All formulations were observed under 40× magnification, and the diameters of microparticles were measured using dedicated image analysis software (Motic Images Plus 3.0) to determine the average particle size for each formulation.

#### 2.3.3. Moisture Presence

The moisture content of the developed formulations was determined using a Radwag WPS 50SX moisture analyzer (Warsaw, Poland). For this purpose, 20 mg of each formulation was placed in an aluminum sheet and heated in the temperature range of 30 to 120 °C.

### 2.4. In Vitro POS Release

According to the European Pharmacopoeia 11 [40], a release testing apparatus (Erweka Dissolution tester type DT 600HH, Heusenstamm, Germany) was applied for the in vitro release profile assessment. In the first stage of the release study, the amount of microparticles corresponding to 20 mg of POS was placed in baskets and immersed for two hours in 375 mL of 0.1 M HCl (pH 1.2), representing gastric conditions. Subsequently, in phase 2, 125 mL of phosphate buffer was added, and the pH was adjusted to 6.8 using 20% sodium hydroxide solution. In the second phase, 1% SDS was added to provide sink conditions. Samples were collected at time points of 30, 60, 90, and 120 min of the first stage, and after 15, 30, 45, 60, 90, 120, 150, and 180 min of the second phase, and then analyzed spectrophotometrically (Section 2.9). Formulations that fulfilled the pharmacopoeia criteria for gastro-resistant drug forms were subjected to further studies.

### 2.5. Mathematical Modeling of the Release Profile

To evaluate the POS release mechanism, the results from the POS release test were assessed using the following mathematical models:

Zero-order kinetics:

F = k × t(4)

First-order kinetics:

lnF = k × t(5)

Higuchi model:

F = kt^1/2^ (6)

Korsmeyer–Peppas model:

F = kt^n^ (7)

Hixson–Crowell model:

1 − (1 − F)^1/3^ = kt (8)

where F is the released drug, k is the constant related to the drug release, and t is the time.

### 2.6. Assessment of Microparticle Morphology

A scanning electron microscope (SEM) with high vacuum mode and secondary electron detector (InspectTMS50, FEI Company, Hillsboro, OR, USA) was employed to evaluate the morphology and shape of the prepared EUD/ALG, EUD, and ALG microparticles. Before analysis, microparticles were covered with a 6 nm gold layer. SEM analysis was conducted under 10 kV voltage and 10 mm of detector working distance. The microparticles were observed under magnifications of 2000×, 5000×, 10,000×, and 20,000×.

### 2.7. Swelling Study

Two different swelling tests were carried out to evaluate the swelling behavior of the formulations under distinct conditions. A method that utilized baskets from the dissolution apparatus was employed to determine the total swelling ratio in an excess of medium. Additionally, Franz diffusion cells were employed for the selected formulations to estimate swelling under restricted fluid access through a semi-permeable membrane, mimicking the condition of a limited amount of medium in the gastrointestinal tract.

#### 2.7.1. USP Dissolution Apparatus Basket Method

The swelling capacity was determined using 0.1 M HCl (pH = 1.2) or phosphate buffer (pH = 6.8). An amount of 20 mg of microparticles was placed in the baskets of USP dissolution equipment and situated in Petri dishes with 20 mL of medium. The test was performed at room temperature (22 ± 2 °C). After specific time intervals, the baskets were taken out, carefully drained, and then weighted using an analytical balance. The swelling ratio (SR) was calculated using the following expression:

SR = (W_S_ − W_0_)/W_0_ (9)

where W_0_ is the microparticle initial weight, and W_S_ is the weight of the swollen microparticles. The swelling capacity study was performed in triplicate.

#### 2.7.2. Franz Diffusion Cells

Formulations in the amount of 10 mg were placed on the regenerated cellulose membrane (0.45 μm pore size) covering the membrane surface. The receiver chamber was filled with 0.1 M HCl or phosphate buffer (pH 6.8), and then thermostated at 37 ± 1 °C. At the set time intervals (after 5, 10, 15, 30, 45, 60, 75, 90, 105, and 120 min), the receiver chamber was refilled with the medium up to the primary level, which was reduced due to the uptake. A graduated microliter syringe (Hamilton, Bonaduz, Switzerland) was applied to refill the medium. The swelling ratio was calculated as the volume of the medium absorbed by one milligram of the formulation [41].

### 2.8. Mucoadhesion Assay

Mucoadhesive properties were evaluated with the use of a TA.XT Plus Texture Analyzer (Stable Micro Systems, Godalming, UK). Porcine gastric and small intestine mucosa were applied as mucoadhesive layers. An amount of 50 mg of each formulation was placed in a sample holder to prevent any loss or scattering of the formulation during the measurement, which was moisturized with 100 µL of the respective medium (0.1 M HCl or phosphate buffer), and shortly mixed to ensure uniform hydration. Fragments of the appropriate mucosa were attached with an adhesive to a cylindrical probe (diameter of 7 mm). Prepared formulations were exposed to contact with the mucosa with a 0.5 N force for 120 s. A cellulose disc was utilized as the negative control. The mucoadhesive properties were assessed by measuring the detachment force (F_max_), and the work of mucoadhesion (W_ad_) value was registered using Texture Exponent 32 Software, version 5.0.

### 2.9. Spectrophotometric Analysis

The analysis was carried out with a spectrophotometer (Jasco V-750, Tokyo, Japan) at a wavelength of 254 nm. The standard calibration curve was linear over the range of 1–50 g/mL and characterized by correlation coefficients (R^2^) of 0.9968 and 0.9995 (0.1 M HCl and phosphate buffer, respectively); the limit of detection (LOD) and limit of quantification (LOQ) were 0.55 µg/mL and 1.65 µg/mL.

### 2.10. Antifungal Activity

To evaluate the antifungal activity of the prepared microparticles, the plate diffusion technique was utilized. Fungus inoculum was prepared with sterile 0.9% NaCl solution, reaching a final density of 5 × 104 CFU/mL (corresponding to 0.5 on the McFarland scale) [42], and then seeded on Petri dishes with Sabouraud dextrose agar (SDA) in the amount of 50 µL. After drying, an amount of the tested formulations corresponding to 5 mg of POS was immersed in 20 µL of 0.1 M HCl and placed in the center of agar plates. POS/DMSO solution was used as the control. The plates with the samples were incubated at 37 ± 1 °C for 24 and 48 h. After this time, the growth inhibition zones were determined by applying a caliper (Mitutoyo, Kawasaki, Japan) with an accuracy of 0.1 mm.

### 2.11. Attenuated Total Reflectance–Fourier Transform Infrared Spectroscopy (ATR-FTIR)

The ATR-FTIR assay for the pure ALG, EUD, POS, and placebo and drug-containing formulations was performed with the use of a Thermo Scientific Nicolet 6700 FTIR spectrophotometer (Waltham, MA, USA). The data was registered from 4000 to 500 cm^−1^ of the wavenumber range by averaging 32 scans with a resolution of 4 cm^−1^ and normalized against the background spectrum.

### 2.12. Thermal Analysis

Thermal analysis was performed using two methods: differential scanning calorimetry (DSC) and thermogravimetric analysis (TGA). The thermal analysis study was carried out for the unprocessed ALG, EUD, POS, and spray-dried microparticles using a Mettler Toledo Star TGA/DSC unit. In the TGA assay, sample masses ranging from 2 to 4 mg were placed in aluminum oxide crucibles and heated from 50 to 900 °C at a constant heating rate of 10 °C/min under an argon flow rate of 40 mL·min^−1^. An empty crucible served as a reference. In the DSC analysis, aluminum crucibles with 3–5 mg weighted samples were heated from 0 to 300 °C at 20 °C/min under an argon flow; an empty pan was used as the reference.

### 2.13. Statistics

The collected data was evaluated via Statistica 13.3 (StatSoft, Tulsa, OK, USA), utilizing one-way analysis of variance (ANOVA) or a Kruskal–Wallis test. The results achieved were implemented as the mean and standard deviations on the basis of three independent experiments.

## 3. Results and Discussion

### 3.1. Microparticle Characteristics

The characteristics of the designed microparticles are presented in Table 2. There were no significant differences in particle size between the EUD placebo, the ALG placebo, and the blended drug-loaded formulations. The largest particle size was observed for the blended formulations EAP9, EAP12, EAP15, and EAP18 (19.03 ± 6.34, 19.04 ± 2.33, 19.73 ± 3.53, and 19.11 ± 1.98 µm, respectively). Among the EUD drug-loaded formulations, those with higher POS content were characterized by slightly higher particle size. Formulations with an EUD/ALG ratio of 2:1 were characterized by higher particle size compared with other EUD/ALG ratios. Drug loading of the developed microparticles depended on the formulation composition and varied from 11.17 ± 1.73% (EAP13) to 44.09 ± 3.62% (EAP6) among the blended formulations and from 14.04 ± 2.74% (EP7) to 45.82 ± 2.68% (EP4) within single-polymer formulations. Encapsulation efficiency (EE) was registered above 70% in most cases (except the EAP11 (56.74 ± 7.72%) and EAP 13 (67.01 ± 10.35%) formulations). About 80% of the formulations were characterized by EE above 80%. There was no registered EE below 70% among the EUD drug-loaded formulations. In most cases, the EE values correlated in direct proportion to the ALG content, which was particularly noticeable among the blended formulations with 4% EUD. EE values above 100% (107.06 ± 14.27% for EP9 and 107.94 ± 10.29% for AP1) may result from the selective deposition of the polymer matrix on the internal surfaces of the spray dryer, likely due to the thermoplastic properties of EUD and the moisture-induced stickiness of ALG. This phenomenon may lead to a partial loss of the matrix during the process. Process yield values were registered in the range of 36.82 ± 3.59% for EAP18 to 91.49 ± 3.42% for EP6. Over 90% of the formulations were characterized by a process yield above 50%, and approximately 50% of the formulations exhibited over 70% process efficiency. Production yield values were higher for EUD drug-loaded formulations compared with blended POS-containing formulations. Moisture content varied from 6.60 ± 0.26% for the EAP12 formulation to 22.36 ± 3.24% for the E3 formulation.

### 3.2. In Vitro POS Release Study

**Figure 1:**
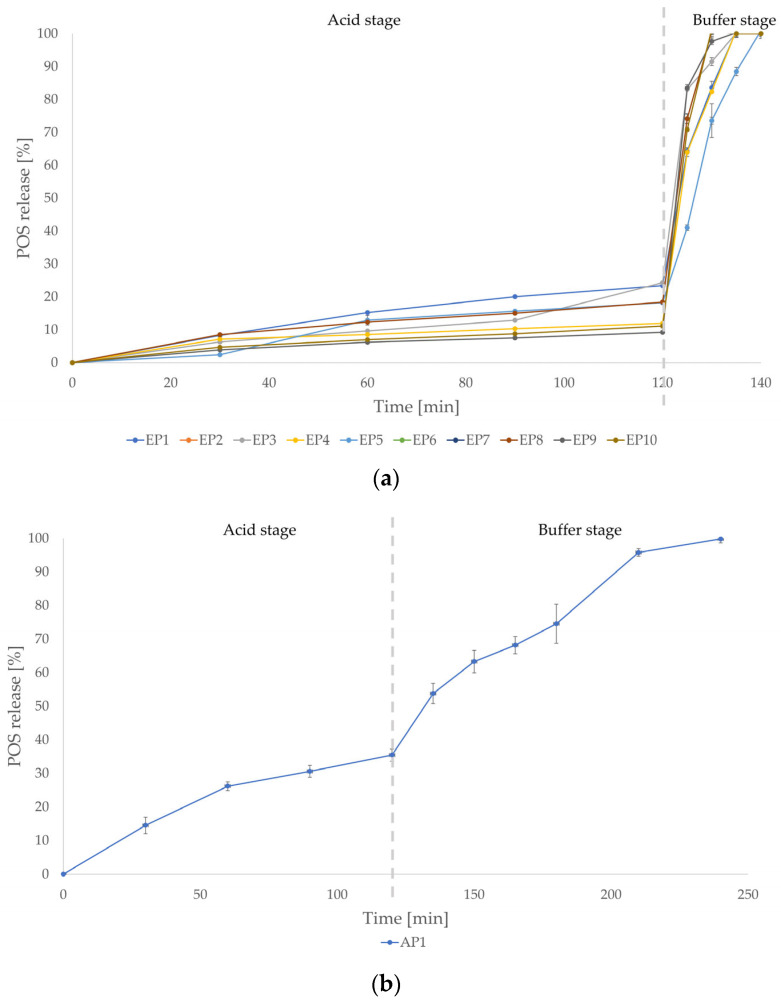
In vitro POS release from the EP1–EP10 formulations (**a**) and AP1 formulation (**b**) (mean ± SD, *n* = 3).

The in vitro drug release assay was conducted in 0.1 M HCl (pH = 1.2) and in phosphate buffer (pH = 6.8), imitating gastric and intestinal conditions, respectively. Phase 1 of the release study was conducted for 2 h in 0.1 M HCl to assess gastrointestinal drug protection. During phase 2, the flasks were refilled with phosphate buffer solution, the pH was adjusted to 6.8, and the study was carried out for a further 3 h. According to the European Pharmacopoeia 11, the permitted amount of drug released from an enteric drug form during the 120 min of phase 1 cannot transcend 10% of the total amount [40]. Since the aim of the study was to develop a gastrointestinal carrier for POS, formulations that did not fulfil these requirements were excluded from further studies. After 120 min of the release test in 0.1 M HCl among the drug-loaded single-polymer formulations (EP1–EP10 and AP1), the amount of POS released was in the range between 9.24 ± 0.26% for EP9 and 35.45 ± 0.38% for AP1 (Figure 1). Only the EP7 and EP9 formulations (1% POS concentration) complied with pharmacopoeia criteria, since they were characterized by POS release under 10% (9.24 ± 0.26% and 9.55 ± 0.27%, respectively). In comparison, the EP8 and EP10 formulations showed a limitation in enteric protection due to the higher drug loading (5% and 6% POS, respectively), which may result in an insufficient polymer/drug ratio. In consequence, a certain amount of the drug remained on the microparticle surface and dissolved rapidly. In phase 2 of the assay, all drug-loaded single-polymer formulations (EP1-EP10 and AP1) released 100% of the POS in the time range of 10 (EP8 and EP10) to 20 min (EP5 and EP7). Only the AP1 formulation was characterized by more sustained drug release compared with the EP1–EP10 formulations—complete POS release was observed after 120 min of phase 2.

**Figure 2:**
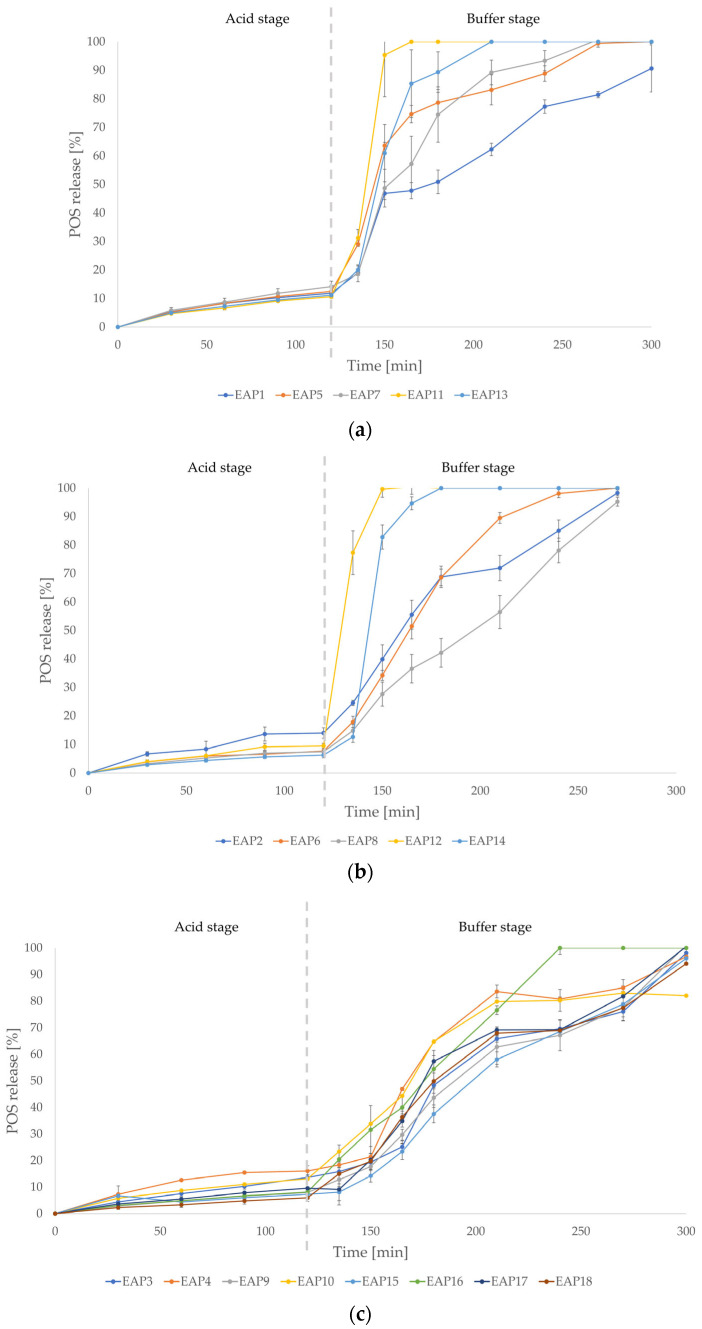
In vitro POS release from the blended EAP1–EAP18 formulations (mean ± SD, *n* = 3) ((**a**)—POS 1%, (**b**)—POS 2%, 3% and 4%, and (**c**)—high ALG content).

Among the blended formulations, EAP1-EAP18, nine fulfilled the pharmacopoeia release criteria for gastrointestinal drug forms (EAP6, EAP8, EAP9, EAP12, and EAP14-EAP18) (Figure 2). None of the blended formulations with EUD in the concentration of 2% (EAP1–EAP4) fulfilled the pharmacopoeia criteria for enteric drug forms. Compared with the EP1–EP10 formulations, where rapid POS release in phase 2 was observed, POS release was more prolonged (up to 2–3 h). The fastest POS release in phase 2 was registered for blended formulations with an EUD/ALG ratio of 4:0.5 EAP11 and EAP12 (POS concentration 1% and 4%, respectively), which were characterized by 100% drug release after 45 and 30 min of phase 2, accordingly. Among the blended formulations with EUD in 3% concentration, EAP5 and EAP7 were characterized by prolonged POS release, although with a “burst effect” between 15 and 30 min and 45 and 60 min of the buffer phase, respectively. The EAP6 and EAP8 formulations possessed more sustained POS release in the intestinal environment. The POS release profile for the EAP9 and EAP10 formulations was prolonged with the rapid increase in released drug between 45 and 60 min of the buffer phase. POS release from the EAP5–EAP10 formulations occurred within 2 to 3 h of phase 2. POS released completely from the EAP13 formulation within 210 min of the release assay, although a rapid increase in POS concentration in the acceptor medium was observed between 15 and 30 min of phase 2. More sustained drug release was observed for the EAP15–EAP18 formulations. Among these, EAP16 was characterized by complete POS release after 120 min of phase 2, with EAP15, EAP17, and EAP18 demonstrating complete release after 180 min. The POS release profile of the blended EUD/ALG formulations varied from the drug release profiles of the ALG and EUD formulations.

The microparticle composition affected the POS release profile. As the EUD concentration in the blended formulations increased, the amount of POS released in 0.1 M HCl decreased. The same was observed with the increased ALG content. The POS content also influenced the drug release in the acidic phase. Among the blended formulations with 3% and 4% of EUD (EAP5–EAP18), formulations with higher POS content (3% and 4%) were characterized by lower drug release after 120 min in 0.1 M HCl compared with formulations with 1% of POS concentration. Despite the fact that the ALG and EUD polymers are insoluble at pH < 3, the majority of the designed single-polymer microparticles AP1 and EP1–EP10 were characterized by higher than 10% POS release in acidic medium after 120 min. Half of the designed blended formulations fulfilled the mentioned criteria. It was observed that formulations with a higher ALG content were more likely to provide enteric protection, which may be related to the swelling process. The results of the swelling study showed a higher swelling ratio of blended POS-containing formulations in an acidic environment compared with EUD drug-loaded formulations. ALG was characterized by higher swelling and gelling ability compared with EUD. ALG in combination with poorly swelling EUD formed a gel matrix restricting diffusion and sealing microparticles, mitigating drug release in the first phase of the assay.

After adjusting the pH to 6.8, significant differences in POS release were reported. A rapid drug release, known as the “burst effect,” was noticeable for all EUD drug-loaded formulations, EAP11, EAP12, and EAP14, and among the blended formulations, and this effect did not occur for AP1. The formulations containing ALG in the microparticle matrix, AP1, and the blended formulations EAP1–EAP18 possessed more sustained POS release. The POS release profile appeared more sustained in formulations with a higher ALG content. The differences detected may be due to disparities in the physicochemical properties of both polymers, which were also observed in the swelling study. The AP1 formulation possessed a significantly higher swelling ratio in phosphate buffer compared with the EUD drug-loaded formulation. Therefore, in phase 2 of the study, the AP1 formulation swelled and formed a gel structure, restricting diffusion and consequently prolonging POS release. This phenomenon did not occur for EUD formulations. The higher POS content may also contribute to prolonging the drug release due to the increased viscosity of the formulation. Taking into account the enteric protection in the acid stage of the release study, and the extension of POS release in the buffer stage, the most promising and optimal systems proved to be the EAP15–EAP18 formulations, since they provided delayed, and the most prolonged, drug release.

The results received from the release assay were applied to mathematical modeling utilizing various mathematical equations for certain release models, such as zero-order kinetics, first-order kinetics, the Higuchi model, and the Korsmeyer–Peppas and Hixson–Crowell models (Table 3) [43]. The highest values of correlation coefficient (R^2^) among the EP1-EP10 formulations were observed for first-order kinetics, indicating the concentration-dependent POS release. The lowest correlation coefficient values were noted in the Hixson–Crowell model. The AP1 formulation exhibited a different POS release profile, correlating the most with the Higuchi model, indicating a drug release profile according to Fickian diffusion. Considering the value of the diffusion exponent (*n*), POS release from the AP1 formulation was not concentration-dependent. Blended formulations exhibited high correlation coefficient values for zero-order kinetics and first-order kinetics with only minor differences, although high values of diffusion exponent (*n*) indicated concentration-independent and non-Fickian POS release. Blended formulations, especially EAP1–EAP3, EAP9, and EAP15–EAP18, possessed higher correlation coefficient values (R^2^) compared with the EUD formulations EP1–EP10.

### 3.3. SEM Analysis of Microparticle Morphology

**Figure 3:**
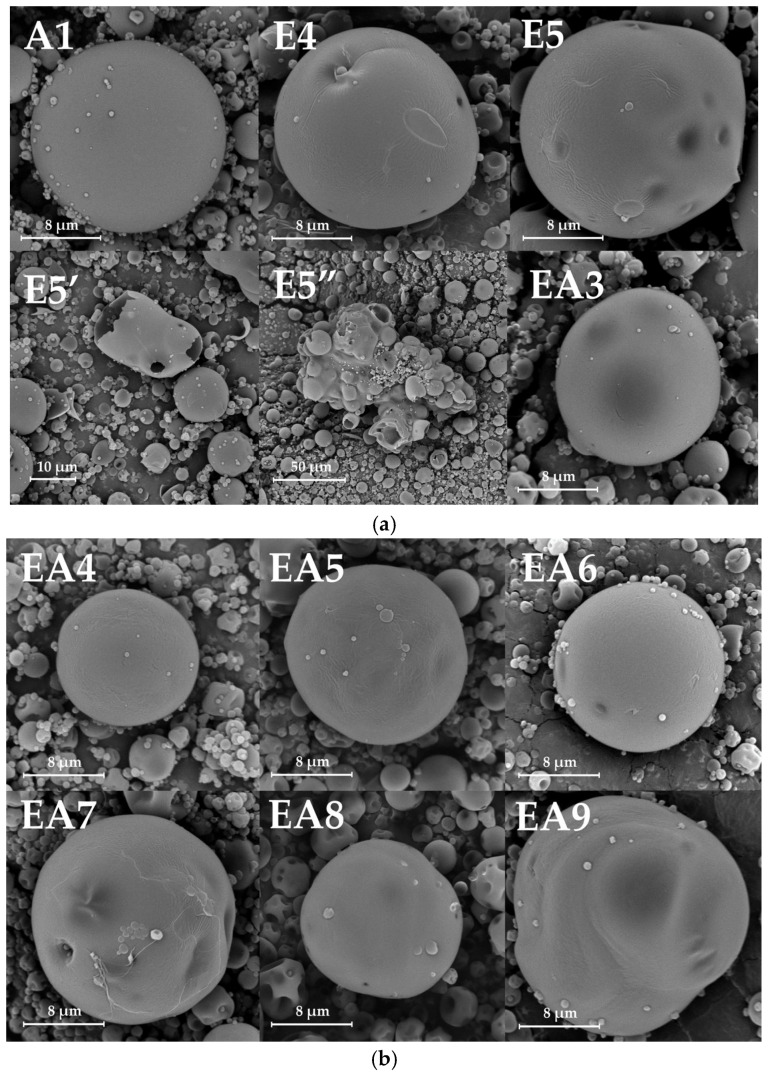

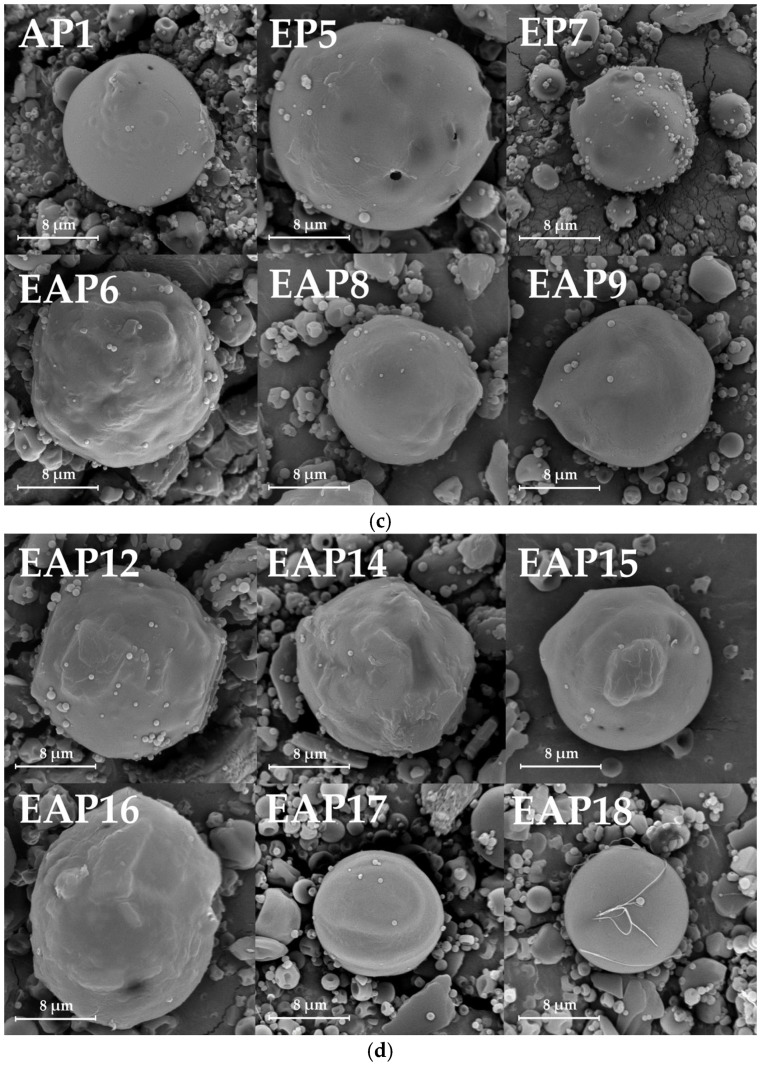
SEM images of placebo (**a**,**b**) and drug-loaded (**c**,**d**) microparticles (E5’—shredded E5 microparticles; E5”—the agglomerates of E5 microparticles).

The morphology of developed microparticles was assessed with the use of SEM technology (Figure 3). The placebo microparticles exhibited a predominantly spherical shape and a smooth, even surface compared with the drug-loaded particles. In the SEM images, the EA7, EA9, and A1 formulations occasionally appeared as microparticles with an irregular shape and rough surface. Microscopic images of EUD placebo microparticles revealed fragmented and shredded microparticles (E5’ in Figure 3a). In addition, agglomerates of microparticles were visible, which were not found in SEM images of other formulations (E5” in Figure 3a). The blended EUD/ALG drug-loaded formulations possessed a spherical shape and a uniform homogeneous surface that was revealed in SEM images. Among the blended formulations, EAP6, EAP8, EAP12, and EAP14 were characterized by a rough and wrinkled surface. Depending on the POS content, differences in the microscopic SEM images of the compound drug-loaded formulations were observed. Blended formulations with lower POS content were characterized by a spherical shape and smooth surface, whereas microparticles of irregular shape with a patchy and wrinkled surface appeared in microscopic images of formulations with higher POS content. In some pictures, minor black marks on the microparticles’ surface were noticeable (EAP15, EAP16, and AP1). The microscopic image of the EUD drug-loaded microparticles differed significantly from blended ones, with more disrupted particles apparent and with more dark stains on the surface (the EP7 and EP9 formulations). In addition, agglomerates composed of multiple microparticles were observed, which were not reported for blended formulations. The surface morphology of the obtained microparticles could be discussed in the context of other EUD/ALG systems, such as those investigated by López et al. [44] and Pastor et al. [45]. The researchers observed predominantly smooth surfaces of EUD microparticles, although blended formulations also exhibited surface roughness. According to their interpretation, temperature-driven desorption led to air nucleation, which ruptured the particle surface. However, it is important to note that in the mentioned studies, significantly different polymer ratios (7.5% EUD and only 0.1% ALG) were utilized, which may alter the viscosity of the feeding liquid and the resulting matrix density.

### 3.4. Swelling Properties

**Figure 4:**
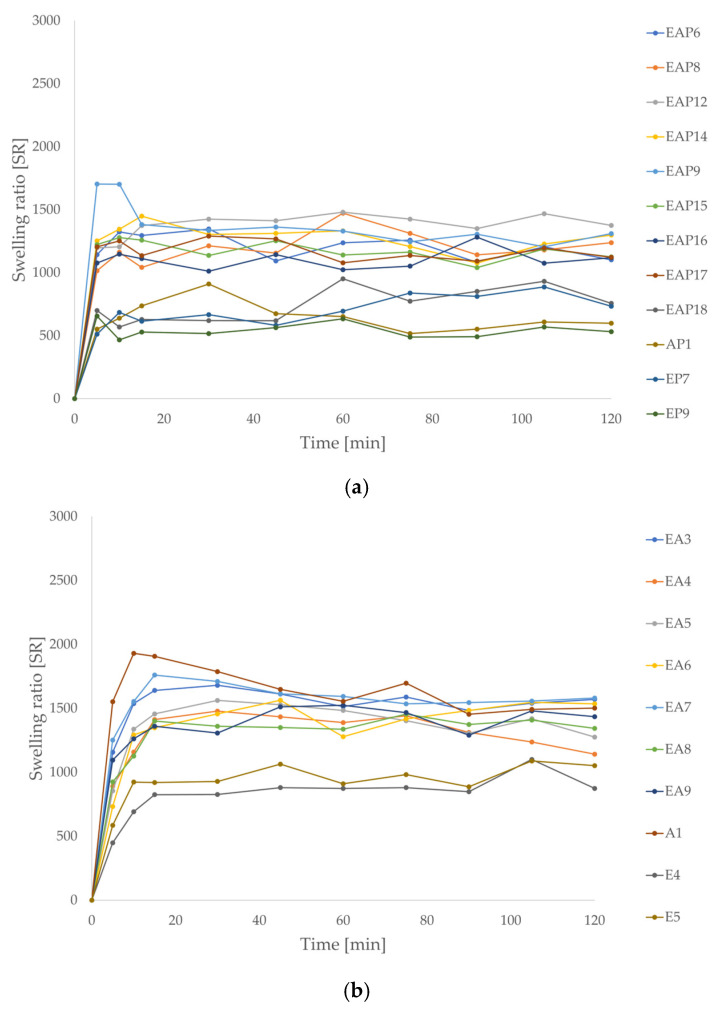
Swelling ratios of POS-loaded (**a**) and placebo formulations (**b**) in 0.1 M HCl (pH = 1.2) (mean ± SD, *n* = 3).

Swelling ability is one of the essential factors influencing the release profiles of drug formulations. The swelling surveys were performed in two environments, 0.1 M HCl (pH 1.2) and phosphate buffer (pH 6.8), imitating gastric and intestinal conditions, respectively. A higher swelling ratio was observed in the phosphate buffer than in hydrochloric acid since at pH 6.8 all carboxylic residues become ionized, which results in electrostatic repulsion of polymer chains and promotes swelling [46]. Drug-loaded formulations were characterized by lower swelling compared with placebo formulations. After 5 to 15 min of the assay in 0.1 M HCl, the particle weight ceased to rise significantly, indicating that water absorption by the microparticles stopped (Figure 4). Among the drug-loaded formulations, the highest swelling properties in 0.1 M HCl were observed for the EAP12 formulation (1375% after 120 min). Blended drug-loaded formulations were characterized by slightly higher swelling ability in gastric conditions (from 1125% for EAP15 to 1375% for EAP12) compared with EUD (from 531.66% to 733.33%) or ALG formulations (598.33%). The polymer applied in the study, Eudragit^®^ L 30 D-55, is an anionic copolymer of methacrylic acid and ethyl acrylate. In an acidic environment (below its pKa 5.5), the carboxyl groups from methacrylic acid undergo protonation, which reduces the negative charge of the polymer molecules, decreasing the forces of mutual repulsion and, as a result, rendering the polymer insoluble in 0.1 M HCl. A similar phenomenon occurs for ALG, which is protonated at pH values below its pKa (3.38–3.65) [12]. Protonation reduced the solubility of both polymers in an acidic environment, which limited the water influx. The EUD/ALG blends showed a higher swelling ratio in placebo and drug-loaded formulations in 0.1 M HCl. The inclusion of ALG to EUD microparticles matrix might cause an increase in swelling in acidic media due to the differences in the physicochemical properties of both polymers. The combination of both polymers may result in the formation of a polyanion network with a greater swelling ability than either polymer individually. A similar phenomenon was observed among placebo formulations. After 10 to 15 min, the particle weight stopped increasing. After the first 15 min, the highest swelling ratio was observed for the A1 formulation. After 120 min, greater swelling ratio values were registered for blended placebo formulations (from 1141.67% for EA4 to 1580% for EA7).

**Figure 5:**
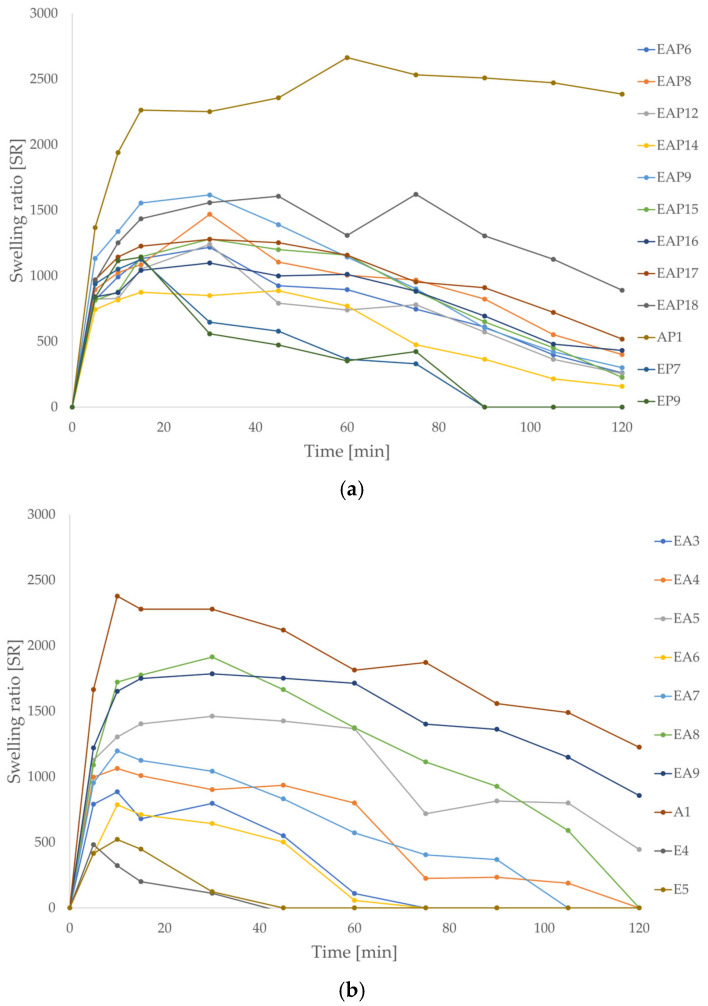
Swelling ratios of POS-loaded (**a**) and placebo formulations (**b**) in phosphate buffer (pH = 6.8) (mean ± SD, *n* = 3).

In the phosphate buffer (pH 6.8), the swelling peak was achieved more slowly than in the acidic medium, after 10 to 45 min (Figure 5). Once the peak was reached, the swelling ratio began to gradually decrease at a rate dependent on the formulation composition. The swelling ratio of the AP1 formulation did not decrease significantly during the 2 h of testing. It was demonstrated that after 120 min of the study, the EP7, EP9, E4, E5, EA3, EA4, and EA6–8 formulations completely dissolved. Mustafine R.I. et al. described a similar phenomenon in their study. They observed weak swelling of pure EUD E PO (butyl methacrylate copolymer, soluble at pH 1–5) during the assay at pH 5.8, while ALG swelled longer and stronger at the same pH [46]. The decrease in the swelling ratio value may be due to the good solubility of both polymers at pH 6.8. Among the blended drug-loaded formulations, the EAP18 formulation exhibited the highest swelling ratio, while EAP14 demonstrated the lowest swelling ratio, after 120 min of the study (890% and 158.33%, respectively). Among the placebo blended formulations, the highest swelling ratio was observed for the EA9 formulation (856.67% after 120 min), and the lowest ratio was for EA5 (786.67% after 10 min, dissolution occurred after 60 min of the study). It was noticeable that among the placebo and drug-loaded formulations, the swelling ratio increased with the increased ALG content. EUD drug-loaded and placebo formulations were characterized by significantly lower swelling ratios compared with ALG and EUD/ALG blended formulations. The swelling ratios of E4, E5, EP7, and EP9 reached the peak earlier than other formulations (about 5 to 10 min) and was characterized by lower peak values. It was visible that POS-containing formulations possessed higher swelling ratios in phosphate buffer (pH 6.8), reached the swelling peak up to 20 min later than placebo formulations, and the swelling process occurred longer. Differences in the physicochemical properties of both polymers may explain the significant differences in the swelling ratio of the blended formulations in the phosphate buffer medium. EUD microparticles exhibited lower swelling compared with the ALG and blended formulations due to good solubility at pH > 5.5 and the lack of gel-forming ability. At pH 6.8, polymethacrylates dissolved, particle mass reduced, and the swelling ratio decreased, whereas ALG swelled, formed a viscous gel structure, and, after a certain period of time, began to dissolve. Compared with the ALG and EUD formulations, EUD/ALG blends were characterized by an increased swelling ratio and a prolonged time required to reach the swelling peak. There is a scarcity of reports in the literature on the pharmaceutical and physicochemical properties of ALG and EUD mixtures. The swelling behavior of the EUD/ALG microparticles reflected the pH-dependent properties of the polymers. A similar relationship was reported by Calija et al. [47] for blends of ALG with EUD L 100–55 (methacrylic acid and ethyl acrylate copolymer, soluble in water at pH >5.5) and oligochitosan, where a higher degree of swelling was recorded in phosphate buffer (pH 6.8) than in simulated gastric fluid (0.1 M HCl). In the acidic environment, the limited swelling of formulations with higher EUD L 100–55 content was linked by the authors to the protonation of the carboxyl groups in ALG and EUD L 100–55, in which the polymers remained insoluble. This correlation between pH and matrix expansion was also observed by Segale et al. [48] in pellets coated with EUD L 100 (methacrylic acid and methyl acrylate copolymer, soluble at pH >6) or EUD L 30 D-55. Notably, after reaching a maximum fluid uptake in phosphate buffer (pH 6.8), a gradual decrease followed due to the erosion and slow dissolution of the polymer matrix. According to Attama A.A., the combination of polymethacrylates with gelatin increased the swelling in water compared with single polymers [49]. Furthermore, Moustafine et al. [46] demonstrated that increasing ALG content enhanced the swelling degree at pH 6.8 for the EUD E PO/ALG polyelectrolyte complex matrix. However, it is important to distinguish that the carrier designed in this work did not concern the formation of polyelectrolyte complexes since ALG and EUD L 30 D-55 possess an anionic character. Prolonged and increased swelling of blended drug formulations in phosphate buffer at pH 6.8 may influence prolonged or sustained POS release in intestinal conditions.

**Figure 6:**
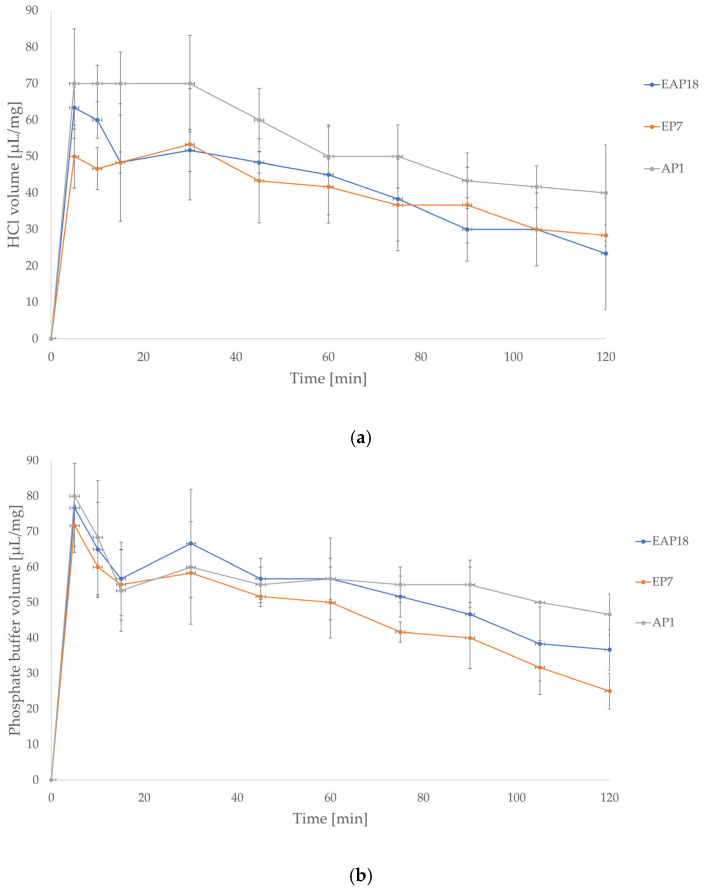
Swelling ratios (SRs) of the EAP18, EP7, and AP1 formulations in 0.1 M HCl (pH = 1.2) (**a**) and in phosphate buffer (pH = 6.8) (**b**) with the use of Franz diffusion cells (mean ± SD, *n* = 3).

The swelling evaluation was also performed with the use of Franz diffusion cells for selected representatives among ALG, EUD, and blended drug-loaded formulations (AP1, EP7, and EAP18, respectively) characterized by the most favorable POS release profiles (Figure 6). The swelling results obtained from the Franz diffusion cells confirmed the observations from the assay performed with the dissolution baskets. In 0.1 M HCl, all formulations reached the swelling peak after 5 min. The AP1 formulation was characterized by a greater quantity of 0.1 M HCl required for refilling the diffusion cell compared with EP7 and EAP18, indicating higher swelling. The EP7 formulation possessed a similar swelling profile to the EP18 formulation, with the exception of the 5 and 10 min periods when the swelling ratio of EP18 was higher than that of EP7. In the phosphate buffer, a swelling peak was observed after 5 min for all formulations, and after 30 min, the swelling ratio began to gradually decrease, which may correlate with the dissolution process beginning to prevail over the swelling. During the first minutes of the assay, the formulations demonstrated comparable swelling rates, after which more pronounced differences in swelling appeared. Comparing the EP7 and EAP18 formulations, it could be concluded that after 30 min of the assay, a higher swelling ratio was registered for EAP18, compared with EP7 since a larger amount of medium was required to refill the Franz diffusion cell. After 30 min, a dissolution of the EP7 formulation in phosphate buffer was observed, which did not occur for the AP1 and EAP18 formulations.

### 3.5. Mucoadhesive Properties

**Figure 7:**
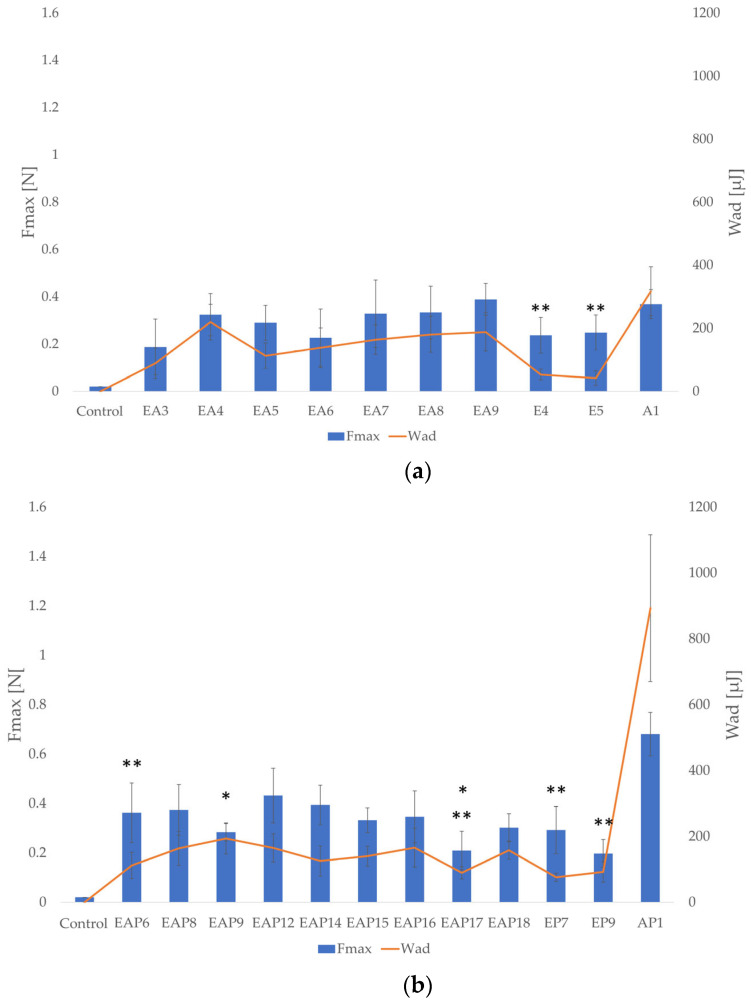
Mucoadhesive properties of placebo (**a**) and drug-loaded (**b**) formulations in 0.1 M HCl (pH = 1.2) (mean ± SD, *n* = 3) (significant statistical difference at (*p* < 0.05) * of F_max_ and ** W_ad_ compared with corresponding values for A1 and AP1).

Mucoadhesion refers to the ability of natural or synthetic materials to adhere to the mucosa surface. Interactions between polymers and mucous membranes involve interactions with mucin, a glycoprotein, which is one of the main components of mucous membranes. This phenomenon includes hydrogen bonding and electrostatic forces. Mucoadhesive properties of a drug form are desirable, since mucoadhesive formulations may remain at the application site longer, increasing the drug bioavailability and reducing the administration frequency. The mucoadhesion study was conducted in simulated gastric and intestinal conditions, with the use of gastric porcine mucosa with 0.1 M HCl and mucosa of porcine small intestine with phosphate buffer at pH 6.8, respectively (Figure 7). The parameters utilized to assess mucoadhesive properties were work of adhesion (W_ad_ [µJ]) and detachment force (F_max_ [N]). All tested formulations exhibited mucoadhesive properties in both environments. In 0.1 M HCl and phosphate buffer, stronger mucoadhesion properties were observed for the drug-containing formulations. In acidic conditions, among the placebo and drug-loaded formulations, the weakest mucoadhesion properties were observed for the EUD formulations (the lowest values of W_ad_ and F_max_). Among the drug-containing formulations, AP1 possessed the highest mucoadhesive properties in 0.1 M HCl (F_max_ = 0.681 ± 0.09 N; W_ad_ = 893.2 ± 222.54 µJ). The highest F_max_ and W_ad_ values within the blended drug-loaded formulations were reported for EAP12 (0.432 ± 0.11 N; 164.98 ± 43.10 µJ, respectively). The EA9 formulation was characterized by the strongest mucoadhesive properties among the placebo formulations in 0.1 M HCl (F_max_ = 0.389 ± 0.07 N; W_ad_ = 188.15 ± 60.69 µJ).

**Figure 8:**
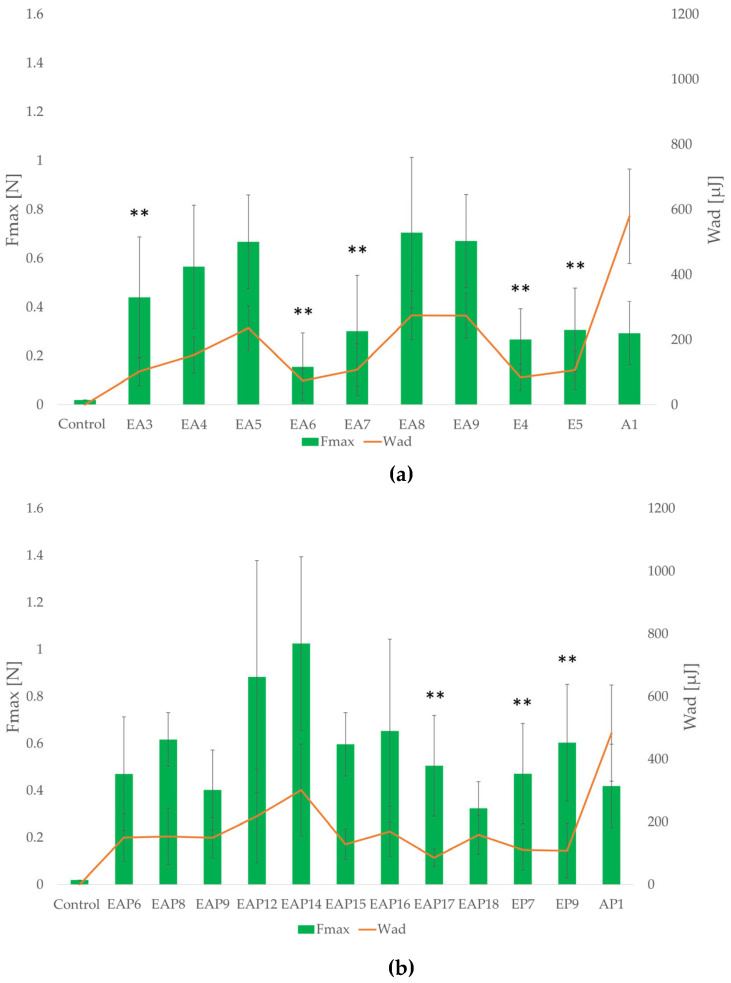
Mucoadhesive properties of placebo (**a**) and drug-loaded (**b**) formulations in phosphate buffer (pH = 6.8) (mean ± SD, *n* = 3) (significant statistical difference at (*p* < 0.05) ** of W_ad_ compared with corresponding values for A1 (placebo formulations) and AP1 (drug-loaded for-mulations)).

All tested placebo and drug-containing formulations showed stronger mucoadhesive properties in phosphate buffer (Figure 8). Among the drug-loaded formulations, EAP14 possessed the strongest mucoadhesiveness (F_max_ = 1.026 ± 0.37 N; W_ad_ = 301.51 ± 147.26 µJ), indicating the potential for prolonged retention at the site of administration, which is a key factor for effective drug delivery. Among the placebo formulations, the EA8 formulation possessed the strongest mucoadhesiveness (F_max_ = 0.706 ± 0.31 N; W_ad_ = 275.00 ± 74.22 µJ). The blended placebo formulations were characterized by stronger mucoadhesive properties compared with the ALG or EUD microparticles, except the EA6 formulation, which possessed the lowest values of the analyzed parameters among all the placebo formulations (F_max_ = 0.156 ± 0.14 N; W_ad_ = 73.99 ± 38.07 µJ).

The values of the tested parameters under acidic conditions increased proportionally with the increasing POS content, which was particularly noticeable among formulations with 4% EUD concentration. The ALG content also affected the mucoadhesive properties. Within the placebo formulations, an increase of F_max_ and W_ad_ values in 0.1 M HCl was observed with the increasing ALG content. However, it was not reported among the blended drug-loaded formulations under the same conditions. In phosphate buffer, the mucoadhesive properties of drug-containing formulations were also improved with increasing ALG content, except those with an EUD/ALG ratio of 2:1. In combination with the drug content, increasing the ALG content may result in excessive viscosity. Prolonging the contact time of the formulation with the mucosa surface is essential for extending the drug release profile. The mucoadhesive performance of the designed formulations is a complex process that is primarily linked with the physicochemical interactions between the polymer matrix and the mucus layer. As noted by Salamat-Miller et al. [50], polymethacrylic acid and its copolymers exhibit a robust ability to form hydrogen bonds with mucin glycoproteins. However, the practical application of pure polymethacrylic acid derivatives is limited by their physicochemical constraints. According to Shojaei [51], the high glass transition temperature (T_g_) of these polymers may result in insufficient chain mobility in the dry state, potentially hindering the initial stages of mucoadhesion by limiting surface wetting and restricting the interpenetration of polymer chains into the mucus layer. To address these limitations, in this study, ALG, as a polymer with well-established and documented mucoadhesive properties [13,14], was blended with EUD. ALG, as a hydrophilic polymer, provided the necessary hydration and structural flexibility to the matrix. The results of this study confirmed that all tested formulations showed mucoadhesive properties in both environments. Since, after oral administration, the drug form passes through the stomach and subsequently the small intestine—the primary site of POS absorption—this study was conducted under simulated gastric and intestinal conditions. Notably, the values of the evaluated parameters were affected by the pH of the medium. Comparing the results of the assay obtained in 0.1 M HCl and phosphate buffer, the formulations demonstrated stronger mucoadhesive properties under intestinal conditions. Given that POS undergoes absorption in the small intestine, extending the formulations’ residence time on the small intestinal mucosa may contribute to prolonged POS release and improved bioavailability. The mucoadhesive potential of polymer blends with various EUD types has been explored across different drug delivery platforms. For instance, Martín-Illana et al. [52] developed bilayer vaginal films consisting of EUD L 100 and chitosan, which demonstrated strong mucoadhesive properties. However, in their study, the polymer ratio did not significantly influence the adhesive strength. In contrast, studies on microparticle drug delivery systems, such as those performed by Kenechukwu et al. [53], showed that EUD RL 100-based (ammonio methacrylate copolymer type A, insoluble in water) microparticles with insulin exhibited high mucoadhesiveness, which was identified by the researchers as a key factor for prolonging contact with the intestinal mucosa and extending the drug release. Furthermore, the integration of EUD L 100 into bilayer patches with gelatin has been reported to provide mucoadhesive properties for buccal delivery, as shown by Maheen et al. [54]. Esporrín-Ubieto D. et al. [55] evaluated various EUD types (RS PO, RL 100, S 100, L 100, and L 100–55) within cross-linked ophthalmic hydrogels. Their findings indicated that the inclusion of EUD S 100 (methacrylic acid–methyl methacrylate copolymer, soluble in water at pH 5–7.5) offered the most favorable mucoadhesive properties. It is worth noting that in studies carried out by López et al. [44] and Pastor et al. [45], ALG (in 0.1% concentration) was added to EUD L30 D-55 microparticles to enhance mucoadhesion. Nevertheless, those reports did not include a quantitative evaluation of the impact of ALG on the mucoadhesive properties of the studied formulations.

### 3.6. Antifungal Activity

**Figure 9:**
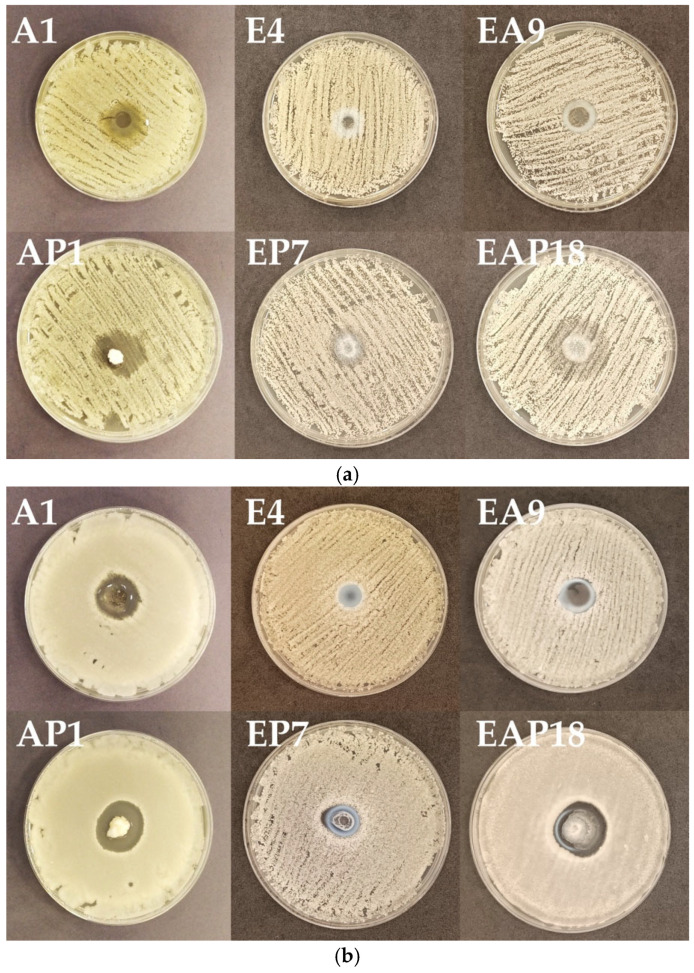

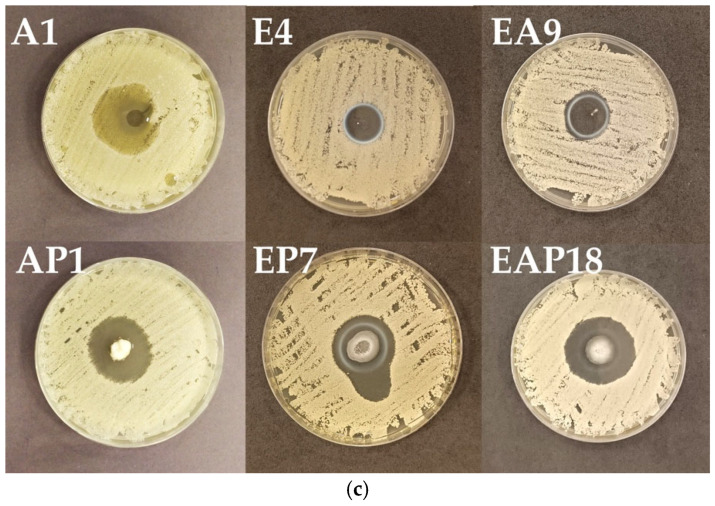
The inhibition zones in the agar diffusion test of drug-loaded formulations characterized by the most favorable POS release profile (AP1, EP7, and EAP18) and placebo formulations (A1, E4, and EA9) against C. *albicans* (**a**), C. *krusei* (**b**), and C. *parapsilosis* (**c**) strains (mean ± SD, *n* = 3).

ALG is known for its biological activity against certain pathogens, although information concerning its antifungal activity is limited [56,57]. Due to its biocompatibility, biodegradability, and non-toxicity, ALG is frequently utilized as a drug carrier for antifungal agents [58,59,60]. ALG may enhance a drug’s antifungal activity by inhibiting the formation of fungal biofilm and potentiate the activity of antifungal drugs [61,62]. There are no reports concerning the antifungal activity of EUD, although there are reports in the literature of drug carriers based on other types of polymethacrylates for antifungal substances, such as films and nanoparticles [63,64,65,66]. Microparticles were subjected to an antifungal activity assay according to the method approved by the Clinical and Laboratory Standards Institute. *Candida* spp. strains were utilized to perform an agar diffusion test [67] (Figure 9).

**Figure 10:**
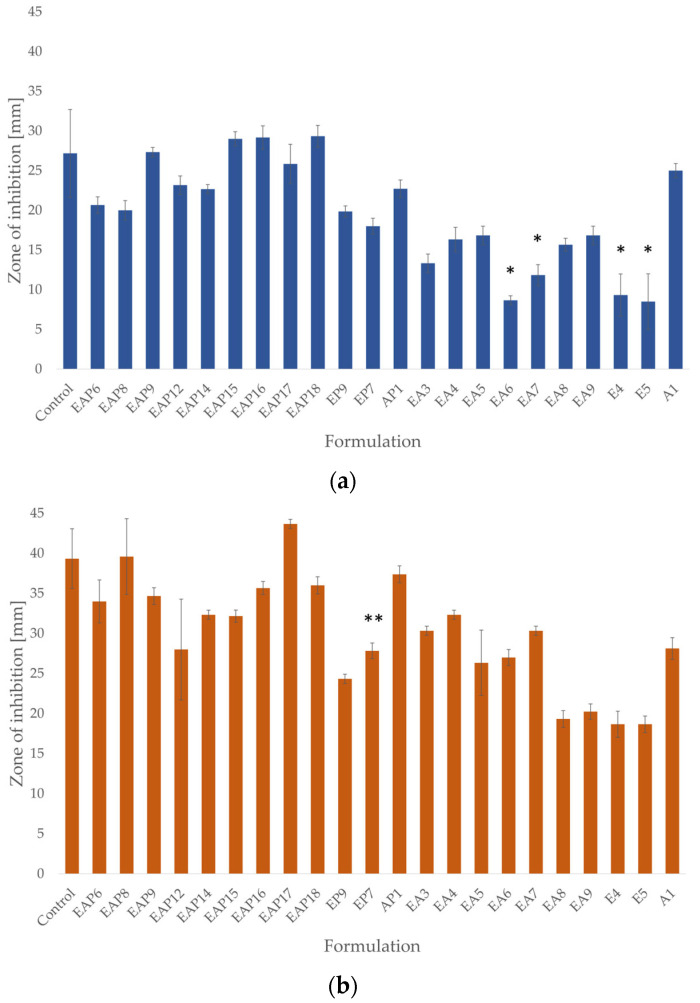

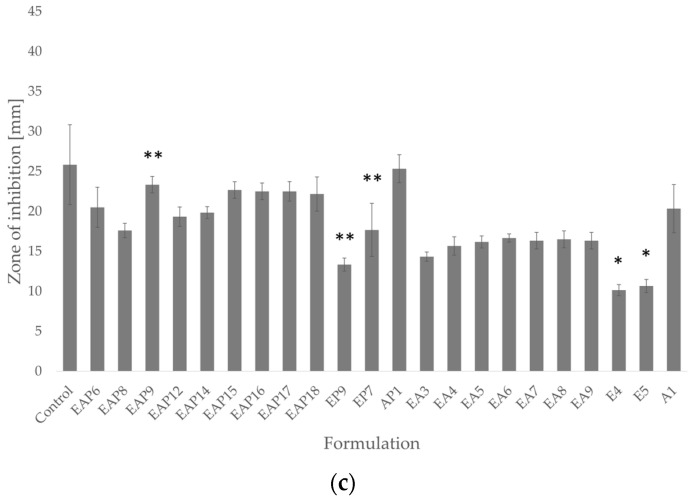
The inhibition zones of drug-loaded and placebo formulations against *C. albicans* (**a**), *C. krusei* (**b**), and *C. parapsilosis* (**c**) (mean ± SD, *n* = 3) (significant statistical difference at (*p* < 0.05) compared with the corresponding values for * A1 and ** AP1).

Drug-containing formulations exhibited stronger antifungal activity compared with placebo formulations (Figure 10). The most sensitive fungi species revealed *C. parapsilosis*, which was characterized by the widest inhibition zones for all formulations tested. The least effective antifungal activity was observed against *C. krusei*. Amongst the tested formulations, the placebo and drug-containing (EP7, EP9, E4, and E5, respectively) EUD formulations demonstrated the weakest antifungal activity against the three tested fungi strains. Among the blended POS-loaded formulations, the strongest antifungal activity against *C. parapsilosis* was exhibited by the EAP17 formulation, while the EAP9, EAP15, EAP16, and EAP18 formulations showed the strongest antifungal activity against *C. albicans.* The EAP9 formulation possessed the highest antifungal activity against the *C. krusei* strain. A modest increase in antifungal activity was noted for the blended drug-loaded formulations compared with the single-polymer formulations AP1, EAP7, and EAP8.

### 3.7. Attenuated Total Reflectance–Fourier Transform Infrared Spectroscopy (ATR-FTIR)

The analysis of the structure of chemical molecules is possible by attenuated total reflectance–Fourier transform infrared spectroscopy (ATR-FTIR). The technique enables the identification of functional groups in molecules and provides insight into the chemical structure of molecules. Moreover, ATR-FTIR spectroscopy enables the identification of potential drug–polymer interactions by assessing the changes, shifts, and intensities in bands of functional groups [68].

**Figure 11:**
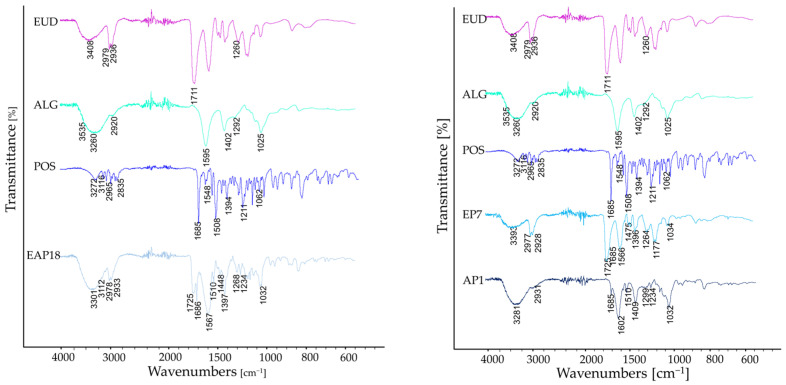
Representative FTIR-AR spectra of the pure polymers ALG and EUD and drug-loaded and placebo formulations.

The FTIR-AR spectra of the studied formulations are shown in Figure 11. In the spectrum of pure ALG (A1), a broad band corresponding to the stretching vibrations of O-H bonds was detected, with a peak at 3260 cm^−1^, along with a signal at 2920 cm^−1^ attributed to the stretching vibrations of C-H bonds. Additionally, bands associated with asymmetric (1595 cm^−1^) and symmetric (1402 cm^−1^) vibrations of the carboxylate group were observed. The spectrum also displayed stretching vibrations of C-O bonds within the pyranosyl ring at 1025 cm^−1^. In the spectrum of the EUD formulations, a broad band around 3410 cm^−1^, which corresponds to stretching vibrations of O-H bonds presented in the carboxylic group of poly(methacrylic acid), was observed. Furthermore, intensive signals around 2980 and 2930 cm^−1^ were detected, corresponding to stretching vibrations of C-H groups presented primarily in the main polymeric chain. A strong signal around 1720 cm^−1^ and a weaker one around 1260 cm^−1^, attributed to the stretching vibrations of C-O bonds presented in ester groups, were also observed.

In the spectra of the blended drug-containing and placebo formulations and the AP1 and A1 formulations, all signals characteristic for ALG were observed; nevertheless, in the formulations with its lower content (EAP6, EAP12, EA3, and EA6), the signals were barely visible and hidden behind the intensive signals of EUD (especially the broad signals attributed to O-H and C=O functional groups). The intensity of signals originating from the functional groups of excipients directly depended on their content in the formulation. The higher the concentration of a given component, the more intense the signals. In addition, the spectra of drug formulations revealed subtle signals indicative of bond vibrations characteristic of POS. Notable among these are the C=O stretching vibration from the urea moiety near 1685 cm^−1^, the aromatic ring stretching observed at 1508 cm^−1^, C-N stretching vibrations at 1394 cm^−1^, aryl-alkyl ether C–O vibrations at 1211 cm^−1^, C–F bond stretching around 1106 cm^−1^, and out-of-plane bending vibrations of the aromatic ring detected at 869 cm^−1^. Similarly, in this case, the intensity of signals originating from the drug depended on its content in the formulation. In formulations with the lowest drug concentration (EAP9, EAP15, EAP17, EP7, and EP8), the characteristic signals of its functional groups were only subtly marked in the spectrum. In contrast, in formulations with the highest drug content (EAP12, EAP14, EAP16, and EAP18), these signals were clearly prominent—especially the signal corresponding to the C=O stretching vibrations (1685 cm^−1^). In the spectra of the EP7, EP8, and AP1 formulations, the characteristic signals from POS functional groups were observed, and no interactions between the excipients and the drug molecule were registered (the signals from POS were not shifted). These findings demonstrate the compatibility between the excipients and the active molecule.

### 3.8. Thermal Analysis

The influence of temperature on the physicochemical properties of the designed formulations was assessed using differential scanning calorimetry (DSC) and thermogravimetric analysis (TGA).

**Figure 12:**
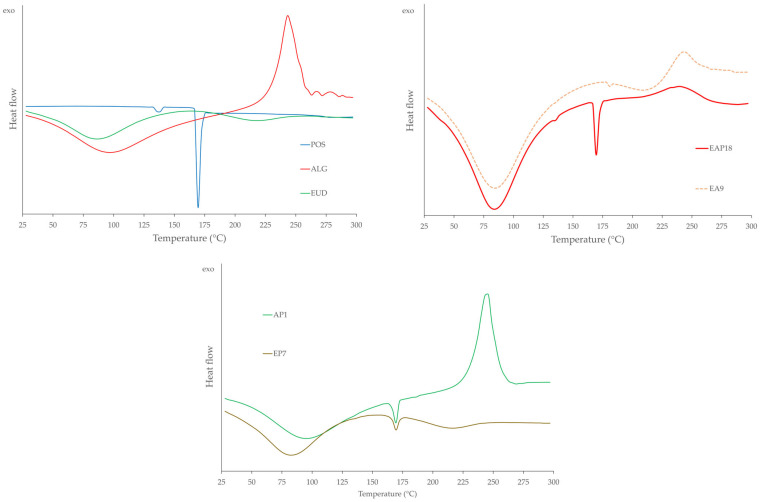
DSC thermograms of the pure polymers ALG and EUD and selected drug-loaded and placebo formulations.

Differential scanning calorimetry (DSC) is a widely employed analytical technique for characterizing the thermal behavior of pharmaceutical formulations. Detailed examination of phase transitions, including the determination of melting points, provides valuable insights into the relationship between the polymeric composition of a dosage form, its microstructural organization, and its physicochemical stability [69,70]. DSC analysis was performed for pure ALG, EUD, POS, blended EUD/ALG, placebo, and drug-loaded formulations (Figure 12). In the thermogram of pure ALG, a broad endothermic peak was observed in the temperature range of 50–190 °C, corresponding to the water evaporation. A sharp exothermic peak with a maximum at 243 °C was also observed, which is likely related to the degradation of the polymer [71]. The thermogram of pure EUD revealed a broad endothermic peak in the range of 50–160 °C, attributed to water loss, and a minor one between 180 and 250 °C, suggesting the onset of polymer thermal decomposition [72,73]. In the DSC thermogram of pure POS, two endothermic peaks were registered: a minor peak at 138 °C and a more intense one at 170 °C. As no mass loss was observed in the corresponding TG thermograms at these temperatures, these events may be attributed to the melting of two different crystalline forms of POS [74]. For the blended formulations, a broad endothermic peak between 50 and 190 °C was observed that was more pronounced than those of the individual polymers, which was likely due to the combined water evaporation from ALG and EUD. An exothermic peak at 243 °C attributed to ALG degradation was also present in thermograms of the blended formulations. The thermograms of all drug-loaded formulations showed two peaks characteristic of POS with no significant shifts in most samples. Formulations containing higher POS content exhibited more distinct drug-related peaks. In the thermograms of the placebo (EA6, EA7) and drug-loaded formulations (EAP12, EAP14), a noticeable flattening and broadening of the exothermic peak associated with the ALG chain decomposition was observed. This behavior may indicate an interaction between EUD at a concentration of 4% and ALG at concentrations of 0.5% and 1%.

**Figure 13:**
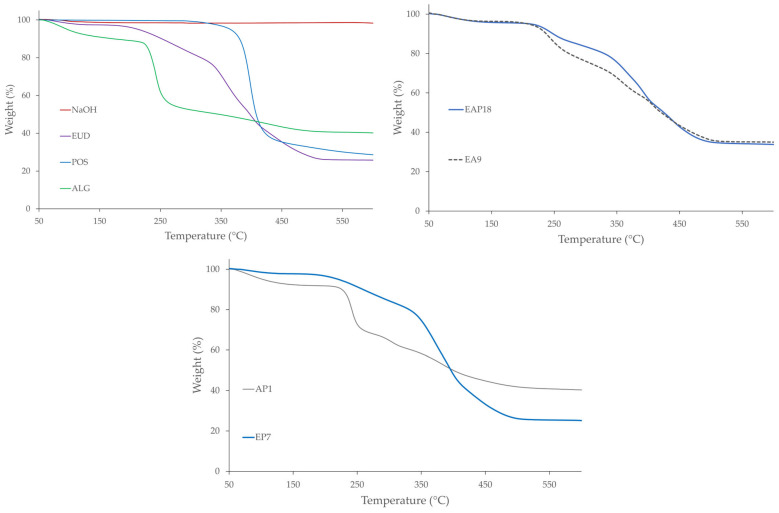
TG thermograms of the pure polymers ALG and EUD and selected drug-loaded and placebo formulations.

**Figure 14:**
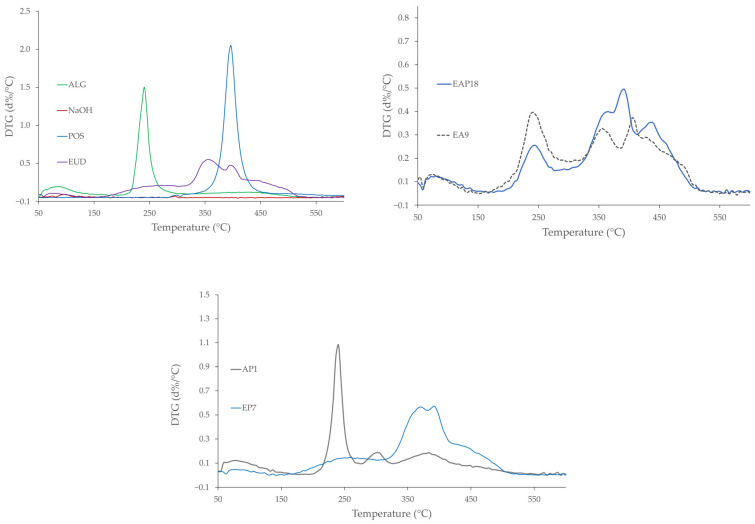
DTG thermograms of the pure polymers ALG and EUD and the selected drug-loaded and placebo formulations.

Thermogravimetric analysis (TGA) is a technique that measures changes in a sample mass as a function of temperature, providing insights into physical processes such as phase transitions, absorption, and desorption, as well as chemical phenomena such as thermal degradation [75,76]. TGA and DTG results revealed a multi-stage thermal degradation for all formulations, except POS, which exhibited a one-step degradation (Figure 13 and Figure 14). The first mass-loss stage in all systems, occurring between 50 and 180 °C, could be ascribed to the removal of water. In the TGA profile of pure ALG, three distinct degradation stages were identified. The first stage (from 50 to 200 °C) accounted for approximately 11% mass loss. The most pronounced degradation occurred in the second stage, with a maximum degradation rate at 236 °C and a mass loss of 37%. The total mass loss observed at 550 °C for ALG was 59%. Pure EUD also revealed a three-step thermal degradation profile, with a total mass loss of 73%. In the first stage (50–150 °C), a minor 3% weight loss was observed. The second stage, with a maximal degradation rate at 271 °C, resulted in 16% weight loss. The third step, which occurred between 310 and 550 °C with two distinct maxima in the degradation rate at 358 and 397 °C, revealed a weight loss of 55%. The latter two stages were associated with the decomposition of the polymethacrylate-based polymer backbone. The TG thermograms of POS-loaded formulations showed that thermal degradation in the second stage started at a higher temperature compared with pure ALG, but at a lower temperature than pure EUD. The majority of the blended, drug-loaded, and placebo formulations exhibited four-step thermal degradation profiles. In the initial step (between 50 and 180 °C), a slight weight loss (from 2% to 4%) was observed. In the second stage (180–310 °C), weight loss ranged from 10% (EAP12) to 27% (EA5). The third stage was characterized by the highest weight loss for the blended formulations (from 36% for EAP6 to 70% for EA12). The percentage of weight loss decreased slightly with the increasing ALG content in the blended formulations. The EUD-based formulations were characterized by the highest total weight loss among all the drug-loaded samples (75% and 73% for EP7 and EP9, respectively) and placebo formulations (74% and 73% for E4 and E5, respectively). In contrast, the ALG-based formulations showed the lowest total weight loss in the placebo (59% for A1) and drug-loaded systems (59% for AP1). The POS content did not affect the TGA thermograms.

Despite the promising results, this study possesses several limitations. The evaluation was conducted under in vitro and ex vivo conditions; thus, the actual in vivo performance and mucoadhesive strength in the human gastrointestinal tract remain to be further investigated. The blended EUD/ALG matrix exhibited favorable pharmaceutical properties; therefore, long-term stability studies are required.

### 3.9. Conclusions

In this study, a delayed and extended release multi-unit drug delivery system for POS was developed. Blended and single-polymer microparticles with POS were successfully obtained by the spray-drying technique. Among the single-polymer microparticles, only the EP7 and EP9 formulations were characterized by delayed release, although the *“burst effect”* in intestinal simulated fluid was observed. The AP1 formulation possessed prolonged POS release in phosphate buffer; however, it did not provide sufficient enteric protection, and did not fulfil the pharmacopoeia criteria in the release study. The combination of both polymers ensured delayed and prolonged POS release. Specifically, the EAP15–EAP18 formulations demonstrated optimal pharmaceutical properties, including a beneficial drug release profile, significant mucoadhesive potential, and a swelling behavior extending the intestinal residence time. These blended formulations may present a promising platform for the development of a multi-unit drug form for POS with delayed and sustained release. Moreover, they could potentially be incorporated into final dosage forms, such as sachets or capsules, to ensure dose flexibility for specific patient populations; however, further studies are required.

## Figures and Tables

**Table 1:** Composition of designed microparticles.

Formulation	EUD Concentration (*w*/*v*%)	ALG Concentration (*w*/*v*%)	POS Concentration (*w*/*v*%)
A1	-	1.5	-
AP1	-	1.5	0.75
E1	2	-	-
E2	3	-	-
E3	4	-	-
E4	5	-	-
E5	6	-	-
EP1	2	-	1
EP2	2	-	2
EP3	3	-	1
EP4	3	-	3
EP5	4	-	1
EP6	4	-	4
EP7	5	-	1
EP8	5	-	5
EP9	6	-	1
EP10	6	-	6
EA1	2	0.5	-
EA2	2	1	-
EA3	3	0.5	-
EA4	3	1	-
EA5	3	1.5	-
EA6	4	0.5	-
EA7	4	1	-
EA8	4	1.5	-
EA9	4	2	-
EAP1	2	0.5	1
EAP2	2	0.5	2
EAP3	2	1	1
EAP4	2	1	2
EAP5	3	0.5	1
EAP6	3	0.5	3
EAP7	3	1	1
EAP8	3	1	3
EAP9	3	1.5	1
EAP10	3	1.5	3
EAP11	4	0.5	1
EAP12	4	0.5	4
EAP13	4	1	1
EAP14	4	1	4
EAP15	4	1.5	1
EAP16	4	1.5	4
EAP17	4	2	1
EAP18	4	2	4

**Table 2:** Characteristics of the designed microparticles.

Formulation	Particle Size (µm)	Percent Loading (%)	Encapsulation Efficiency (%)	Production Yield (%)	Moisture Content (%)
A1	13.51 ± 1.02	-	-	60.65 ± 12.08	17.66 ± 1.04
AP1	13.95 ± 1.70	35.94 ± 3.68	107.94 ± 10.29	58.72 ± 2.40	18.43 ± 0.57
E1	11.24 ± 1.02	-	-	73.81 ± 6.94 *	20.86 ± 0.87
E2	13.80 ± 4.11	-	-	57.79 ± 8.17	13.62 ± 1.63
E3	13.31 ± 2.13	-	-	79.28 ± 7.73 *	22.36 ± 3.24
E4	13.81 ± 2.36	-	-	87.72 ± 8.43 *	20.32 ± 1.46
E5	13.94 ± 2.01	-	-	76.78 ± 5.17 *	21.45 ± 1.82
EP1	11.77 ± 1.26	27.20 ± 1.76	81.69 ± 5.29	82.93 ± 7.69 *	11.51 ± 0.60
EP2	13.36 ± 2.10	41.32 ± 1.82	82.63 ± 3.63	75.24 ± 7.24 *	12.68 ± 1.23
EP3	13.51 ± 2.65	18.33 ± 2.11	73.33 ± 8.45 *	67.25 ± 6.71	13.92 ± 2.07
EP4	13.40 ± 1.79	45.82 ± 2.68	91.63 ± 5.36	78.89 ± 3.47 *	17.85 ± 0.68
EP5	11.72 ± 1.27	16.52 ± 2.16	82.59 ± 10.81	75.74 ± 2.27 *	14.30 ± 1.49
EP6	13.91 ± 2.58	43.32 ± 3.68	86.64 ± 7.36	91.49 ± 3.42 *	11.71 ± 0.82
EP7	13.73 ± 2.80	14.04 ± 2.74	84.21 ± 16.47	72.04 ± 2.64 *	14.26 ± 0.60
EP8	14.15 ± 2.12	43.85 ± 1.93	87.70 ± 3.86	70.70 ± 4.84 *	18.30 ± 1.14
EP9	14.36 ± 2.79	15.29 ± 2.04	107.06 ± 14.27	74.16 ± 4.47 *	15.54 ± 0.51
EP10	16.05 ± 1.65	43.50 ± 0.81	87.01 ± 1.64	71.94 ± 3.76 *	13.55 ± 1.17
EA1	14.23 ± 1.14	-	-	75.14 ± 5.65 *	9.21 ± 1.27
EA2	10.76 ± 1.09	-	-	71.48 ± 7.11 *	14.58 ± 0.50
EA3	12.27 ± 0.94	-	-	84.19 ± 3.88 *	15.90 ± 1.80
EA4	14.31 ± 1.68	-	-	77.62 ± 2.98 *	14.09 ± 2.24
EA5	14.70 ± 1.70	-	-	74.68 ± 6.57 *	18.84 ± 1.76
EA6	12.75 ± 1.29	-	-	76.08 ± 1.97 *	18.06 ± 0.99
EA7	13.31 ± 1.06	-	-	76.79 ± 5.53 *	12.90 ± 1.47
EA8	15.82 ± 2.54	-	-	47.02 ± 7.84 *	18.25 ± 1.22
EA9	15.93 ± 2.93	-	-	56.02 ± 6.63	18.30 ± 1.14
EAP1	14.75 ± 2.24	24.62 ± 3.45	86.17 ± 12.09	56.19 ± 4.36	10.25 ± 1.38
EAP2	16.47 ± 1.22	37.96 ± 6.05	81.16 ± 5.13	68.69 ± 2.62 *	12.13 ± 1.48
EAP3	15.48 ± 1.47	21.98 ± 1.19	87.91 ± 4.75	71.71 ± 7.41 *	10.70 ± 1.46
EAP4	13.02 ± 2.67	28.08 ± 4.22	70.19 ± 10.55 *	65.10 ± 2.74	13.25 ± 1.28
EAP5	16.24 ± 2.64	16.80 ± 1.41	75.59 ± 6.32 *	61.03 ± 4.13	13.40 ± 1.42
EAP6	16.84 ± 2.28	44.09 ± 3.62	95.53 ± 7.84	70.57 ± 6.11 *	15.64 ± 0.66
EAP7	14.42 ± 3.30	18.60 ± 1.48	93.00 ± 7.40	53.87 ± 2.20	15.70 ± 1.89
EAP8	15.05 ± 3.66	42.22 ± 3.89	98.51 ± 9.08	64.18 ± 1.80	12.17 ± 1.08
EAP9	19.03 ± 6.34	17.16 ± 1.69	94.39 ± 9.30	64.88 ± 2.85	15.32 ± 1.20
EAP10	16.84 ± 4.85	35.02 ± 4.63	87.55 ± 11.57	57.89 ± 5.00	14.88 ± 1.76
EAP11	16.67 ± 2.51	10.32 ± 1.40 *	56.74 ± 7.72 *	58.79 ± 3.78	13.65 ± 0.81
EAP12	19.04 ± 2.33	40.70 ± 1.96	86.49 ± 4.17	69.15 ± 3.26 *	6.60 ± 0.26
EAP13	14.87 ± 2.54	11.17 ± 1.73 *	67.01 ± 10.35 *	43.33 ± 1.67 *	16.33 ± 2.04
EAP14	17.24 ± 2.34	42.48 ± 1.52	95.59 ± 3.42	51.44 ± 5.97	18.32 ± 1.43
EAP15	19.73 ± 3.53	12.33 ± 1.95	80.11 ± 12.64	43.61 ± 2.41 *	14.98 ± 0.29
EAP16	18.21 ± 2.46	36.22 ± 5.05	86.01 ± 12.00	42.37 ± 4.46 *	14.51 ± 0.89
EAP17	18.49 ± 3.62	13.00 ± 1.32	91.03 ± 9.22	54.20 ± 6.58	16.51 ± 1.69
EAP18	19.11 ± 1.98	29.32 ± 2.53	73.30 ± 6.33 *	36.82 ± 3.59 *	19.53 ± 1.18

* Significant difference (*p* < 0.05) compared with the corresponding value of AP1.

**Table 3:** Kinetic modeling of POS release from selected formulations.

Formulation	Zero-Order Kinetics		First-Order Kinetics		Higuchi Model		Korsmeyer–Peppas Model			Hixson–Crowell Model	
	R^2^	K	R^2^	K	R^2^	K	R^2^	K	*n*	R^2^	K
**Blended formulations**											
EAP6	0.88	0.50	0.90	0.02	0.90	21.74	0.82	1.28	1.76	0.78	0.01
EAP8	0.89	0.40	0.95	0.01	0.95	26.01	0.87	1.26	1.67	0.81	0.01
EAP9	0.91	0.39	0.95	0.01	0.95	27.79	0.89	1.26	1.71	0.87	0.01
EAP12	0.71	0.52	0.73	0.03	0.71	17.10	0.79	1.30	1.86	0.73	0.03
EAP14	0.73	0.55	0.78	0.02	0.75	16.53	0.76	1.28	2.04	0.79	0.02
EAP15	0.87	0.37	0.89	0.01	0.90	27.56	0.71	1.23	1.45	0.80	0.01
EAP16	0.91	0.46	0.91	0.01	0.93	24.81	0.89	1.27	1.81	0.80	0.02
EAP17	0.89	0.40	0.91	0.01	0.91	26.67	0.86	1.26	1.64	0.67	0.02
EAP18	0.91	0.38	0.90	0.02	0.93	26.44	0.88	1.26	1.90	0.87	0.01
**Single-polymer formulations**											
EP7	0.70	0.90	0.80	0.03	0.74	9.40	0.67	1.31	2.10	0.47	0.03
EP9	0.70	0.95	0.80	0.03	0.74	9.81	0.68	1.31	2.22	0.47	0.03
AP1	0.96	0.44	0.96	0.01	0.97	31.95	0.93	0.62	0.91	0.87	0.01

## Data Availability

The original contributions presented in this study are included in the article. Further inquiries can be directed to the corresponding author.
